# Enhancing Preoperative Assessment of Endometrial Cancer: The Role of Diffusion-Weighted Magnetic Resonance Imaging in Evaluating Myometrial Invasion

**DOI:** 10.7759/cureus.62111

**Published:** 2024-06-10

**Authors:** Sam Raja, Praveen K Sharma, Sakthi Ganesh Subramonian, Chakradhar Ravipati, Paarthipan Natarajan

**Affiliations:** 1 Radiodiagnosis, Saveetha Medical College and Hospital, Saveetha Institute of Medical and Technical Sciences, Saveetha University, Chennai, IND

**Keywords:** contrast media, neoplasm staging, myometrium, diffusion-weighted magnetic resonance imaging, magnetic resonance imaging, endometrial neoplasms

## Abstract

Background: Endometrial cancer (EC) is the most common gynecological malignancy. Accurate preoperative staging is essential for guiding treatment. The depth of myometrial invasion is a key prognostic factor. This prospective study aimed to evaluate the added benefit of diffusion-weighted imaging (DWI) compared to T2-weighted imaging (T2WI) and dynamic contrast-enhanced MRI (DCE-MRI) for the preoperative assessment of myometrial invasion in EC.

Aim and objectives: The aim of this prospective study was to evaluate the added benefit of DWI in the preoperative assessment of myometrial invasion in EC, in comparison with T2WI and DCE-MRI. The objectives were to assess the imaging characteristics of endometrial carcinoma on T2WI, DCE, and DW MR, to assess the depth of myometrial invasion and overall stage in EC patients, to compare the diagnostic performance of DCE-MRI with that of DW-MRI combined with T2WI, to describe how MR imaging findings can be combined with tumor histologic features and grading to guide treatment planning, and to evaluate the pitfalls and limitations of DCE and DW MR in the assessment of EC.

Materials and methods: Thirty-one patients with histologically confirmed EC underwent preoperative pelvic MRI on a 1.5T scanner. T2WI, DWI (b-values 0, 1000 s/mm^2^), and DCE-MRI were performed. Two radiologists independently assessed myometrial invasion on T2WI, T2WI + DWI, and T2WI + DCE-MRI. Histopathology after hysterectomy was the reference standard. Diagnostic accuracy, sensitivity, specificity, positive predictive value (PPV), and negative predictive value (NPV) were calculated for each MRI protocol, with separate analyses for superficial (<50%) and deep (≥50%) myometrial invasions.

Results: The accuracy for assessing superficial invasion was 61.3% for T2WI, 87.1% for T2WI + DWI, and 87.1% for T2WI + DCE-MRI. For deep invasion, accuracy was 64.5% for T2WI, 90.3% for T2WI + DWI, and 90.3% for T2WI + DCE-MRI. Sensitivity, specificity, PPV, and NPV for T2WI + DWI and T2WI + DCE-MRI were high and comparable (88.9-91.7%) for both superficial and deep invasions. T2WI had markedly lower sensitivity and specificity. The differences between T2WI and the functional MRI protocols were statistically significant (p < 0.01).

Conclusion: DWI and DCE-MRI significantly improve the diagnostic performance of MRI for the preoperative assessment of myometrial invasion depth in EC compared to T2WI alone. DWI + T2WI and DCE-MRI + T2WI demonstrate comparable high accuracy. DWI may be preferable since it is faster and avoids contrast administration.

## Introduction

Endometrial cancer (EC) is the most prevalent gynecological malignancy in developed countries and the sixth most common cancer in women worldwide [1]. The incidence of EC has been increasing, likely due to factors, such as rising obesity rates, decreased use of menopausal hormone therapy, and changes in reproductive behaviors [2]. Although EC is often diagnosed at an early stage, the prognosis varies widely depending on histological subtype and grade, local tumor stage, and the presence of lymph node metastases [3]. Accurate preoperative staging is crucial for guiding treatment decisions and surgical planning in EC [4]. The depth of myometrial invasion is one of the most important prognostic factors, correlating with tumor grade, lymph node metastases, and overall patient survival [5,6]. The updated Federation of Gynecology and Obstetrics (FIGO) staging system incorporates MRI findings, with stage IA representing superficial myometrial invasion (<50%) and stage IB indicating deep invasion (≥50%) [7].

MRI is widely recognized as the most accurate imaging modality for preoperative staging of EC, particularly for assessing myometrial invasion and cervical stromal involvement [8]. Conventional MRI protocols typically include high-resolution T2-weighted imaging (T2WI) and dynamic contrast-enhanced MRI (DCE-MRI) [9]. However, the added value of functional MRI techniques, such as diffusion-weighted imaging (DWI), is an active area of research.

DWI provides information about tissue cellularity and microstructure by measuring the diffusion of water molecules [10]. In EC, the high cellularity of malignant tumors results in restricted diffusion, leading to high signal on high b-value DWI and low apparent diffusion coefficient (ADC) values. Several studies have suggested that DWI may improve the diagnostic performance of MRI for assessing the depth of myometrial invasion in EC. However, more research is needed to establish the added benefit of DWI and its potential to replace DCE-MRI in the preoperative staging of EC.

The purpose of this prospective study was to evaluate the added value of DWI compared to T2WI and DCE-MRI for the preoperative assessment of myometrial invasion depth in patients with EC, using histopathology as the reference standard.

## Materials and methods

This prospective observational study was conducted in the Department of Radiology at Saveetha Medical College and Hospital, Chennai, India. The study protocol was approved by the Institutional Human Ethics Committee, and informed consent was obtained from all patients prior to their participation in the study. The study population consisted of 31 patients with histologically confirmed EC, irrespective of the type, who were referred for preoperative MRI between September 2020 and March 2022. Both pre- and post-menopausal patients were included. Patients with cardiac pacemakers, prosthetic heart valves, cochlear implants, metallic implants, claustrophobia, or a glomerular filtration rate less than 30 ml/min were excluded.

MRI examinations were performed on a Philips Multiva 1.5 Tesla scanner (Philips, Netherlands) using a pelvic phased-array multi-coil with the patient in the supine position. The MRI protocol included T1W sequences in the axial plane; T2W sequences in the axial, sagittal, and coronal planes; DWI with b-values of 0 and 1000 s/mm^2^; and dynamic contrast-enhanced (DCE) MRI after intravenous administration of 0.1 mmol/kg gadolinium at 2 ml/s. DWI was acquired in the axial plane prior to contrast administration using a single-shot echo-planar imaging sequence (TR/TE effective range, 1000/74 ms; slice thickness 4 mm; FOV: 36-42 cm; matrix: 384 x 256).

**Figure 1:**
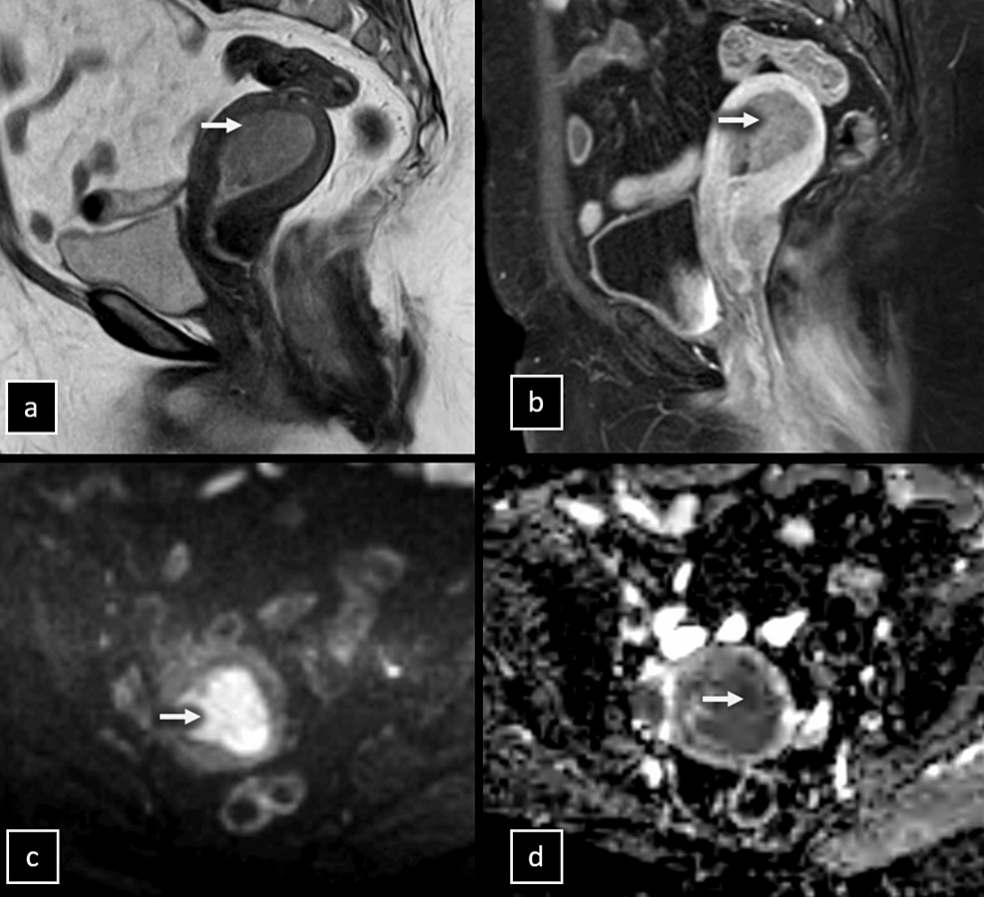
MRI scans of a 72-year-old female patient with stage IA endometrial cancer display. Sagittal T2-weighted image showing a tumor infiltrating the myometrium's outer half (a). Sagittal dynamic contrast-enhanced image depicting the tumor with lower signal intensity relative to the surrounding myometrium, indicating superficial invasion (b). Axial diffusion-weighted image revealing the tumor's high signal at a b-value of 1000 s/mm² (c) and corresponding low signal on the ADC map (d), supporting a diagnosis of superficial myometrial invasion, which was later confirmed by postoperative histopathology.

**Figure 2:**
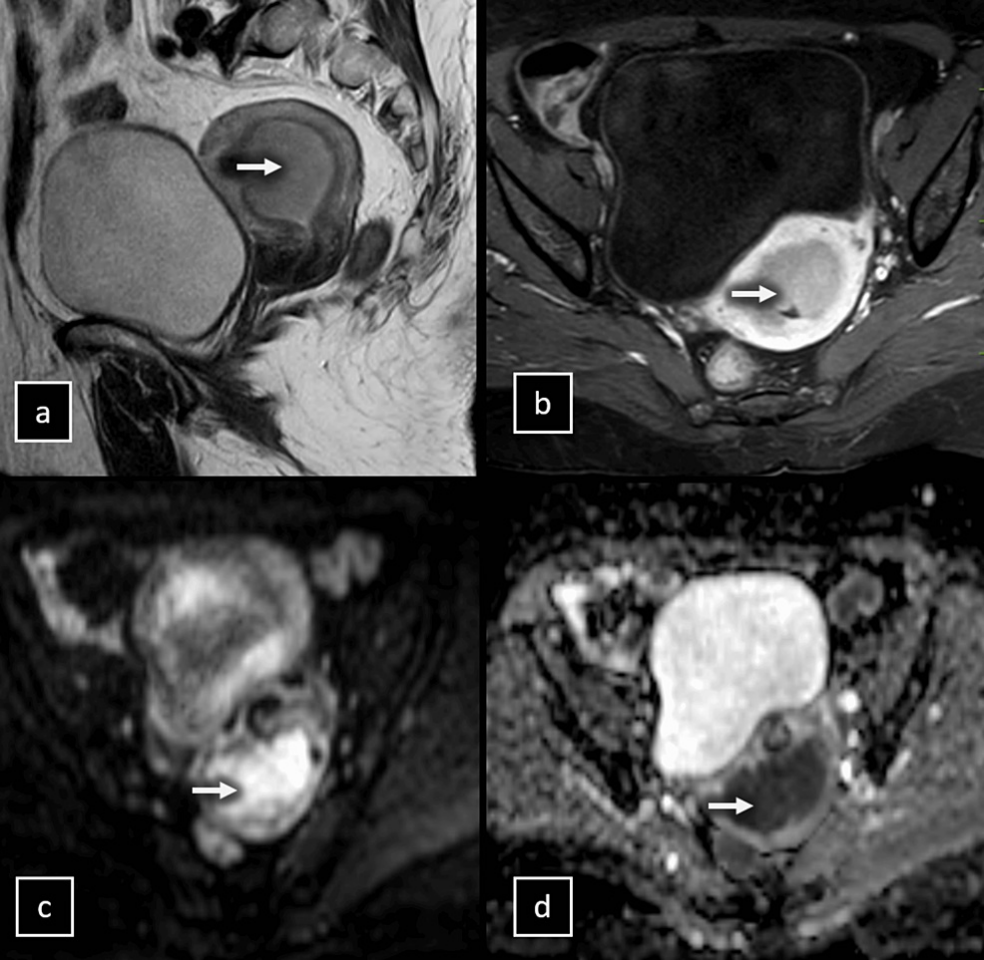
MRI of a 66-year-old woman with endometrial cancer. (a) Sagittal T2-weighted image (T2WI) showing a large endometrial tumor (arrow), initially interpreted as superficial myometrial invasion. (b) Axial oblique dynamic contrast-enhanced MRI (DCE-MRI) sequence at 120 s, perpendicular to the main uterine axis, depicting the large endometrial tumour (arrow) with a hypointense signal compared to the hyperintense myometrium, initially classified as superficial myometrial invasion. (c) Axial diffusion-weighted imaging (DWI) at a b-value of 1000 s/mm^2^ revealing tumor invasion (arrows) into the outer half of the myometrium. (d) Apparent diffusion coefficient (ADC) map confirming the deep myometrial invasion. Postoperative histological analysis confirmed the deep myometrial invasion, staging the tumor as IB.

**Figure 3:**
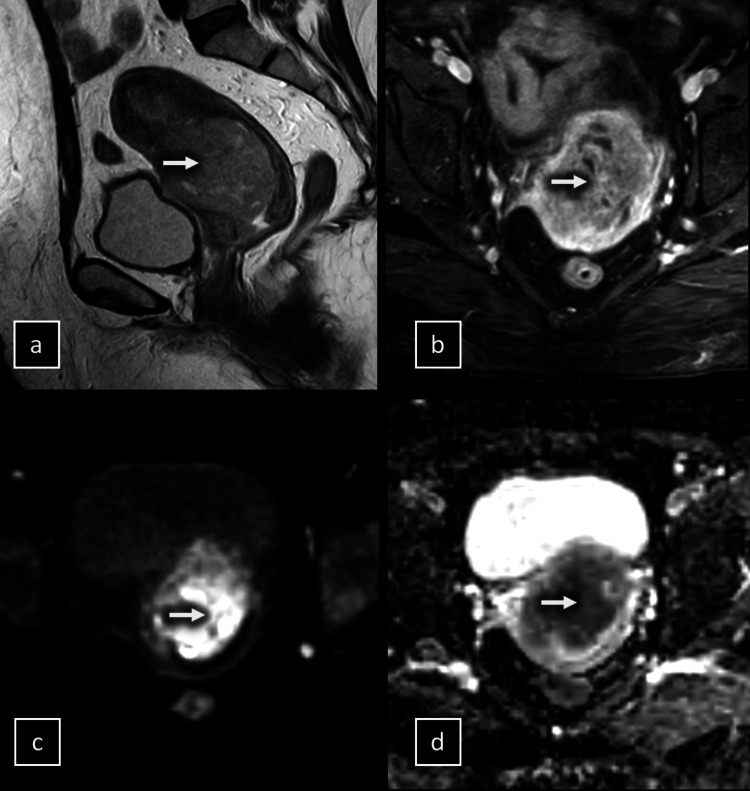
MRI of a 51-year-old woman with endometrial cancer. (a) Sagittal T2-weighted image (T2WI) demonstrating a large endometrial tumor involving the lower two-third of the endometrium and invading more than 50% of the myometrium (arrow), indicative of deep myometrial invasion. (b) Axial oblique dynamic contrast-enhanced MRI (DCE-MRI) sequence at 120 s, perpendicular to the main uterine axis, showing the large endometrial tumor (arrow) with a hypointense signal compared to the hyperintense myometrium, classified by the observers as deep myometrial invasion. (c) Axial diffusion-weighted imaging (DWI) at a b-value of 1000 s/mm^2^ confirming tumor invasion (arrows) into the outer half of the myometrium. (c) Apparent diffusion coefficient (ADC) map further supporting the presence of deep myometrial invasion. Postoperative histological analysis validated the deep myometrial invasion, classifying the tumor as stage IB.

Two radiologists independently assessed the depth of myometrial invasion on T2WI, T2WI + DWI, and T2WI + DCE-MRI. The apparent diffusion coefficient (ADC) values were calculated by placing regions of interest (ROIs) on the ADC maps in areas showing restricted diffusion, avoiding hemorrhage and necrosis. The mean ADC value was calculated as the average of two ROI measurements (Figures 1-3). Surgical histopathology served as the reference standard for assessing the depth of myometrial invasion and overall FIGO stage.

Statistical analysis was performed using IBM SPSS Statistics software for Windows, version 23.0 (IBM Corp., Armonk, NY). Descriptive statistics, including frequency analysis and percentage analysis, were used for categorical variables, while the mean and standard deviation were used for continuous variables. The chi-square test was used to assess the significance of categorical data. Receiver operating characteristic (ROC) curves were used to evaluate the diagnostic performance of the MRI protocols, with sensitivity, specificity, positive predictive value (PPV), negative predictive value (NPV), and accuracy calculated. A p-value of <0.05 was considered statistically significant.

## Results

**Figure 4:**
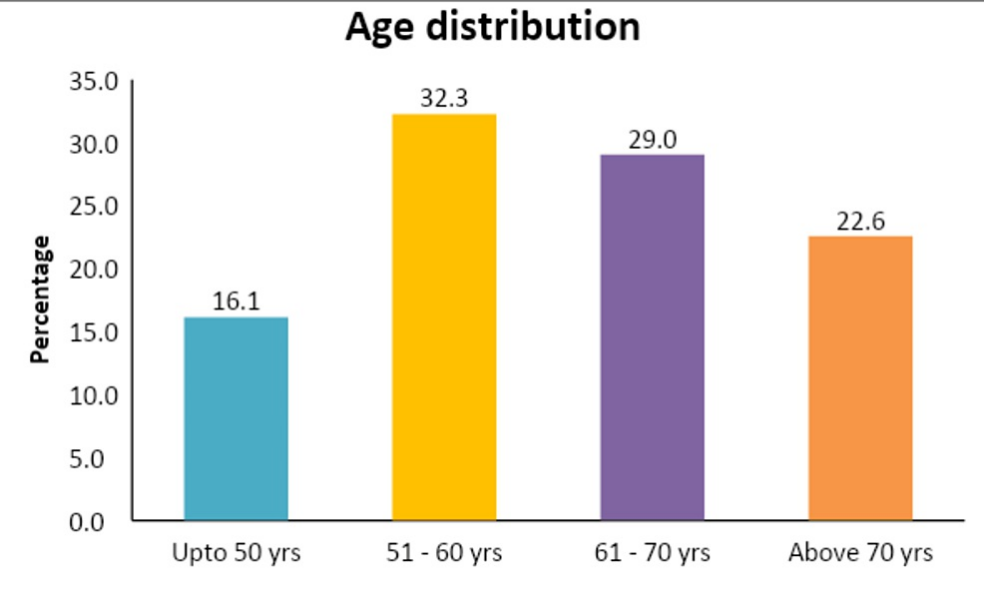
Age distribution of the study population

The study population consisted of 31 patients with histologically confirmed EC. The median age at diagnosis was 60 years (range: 37-81 years) (Figure 4).

**Figure 5:**
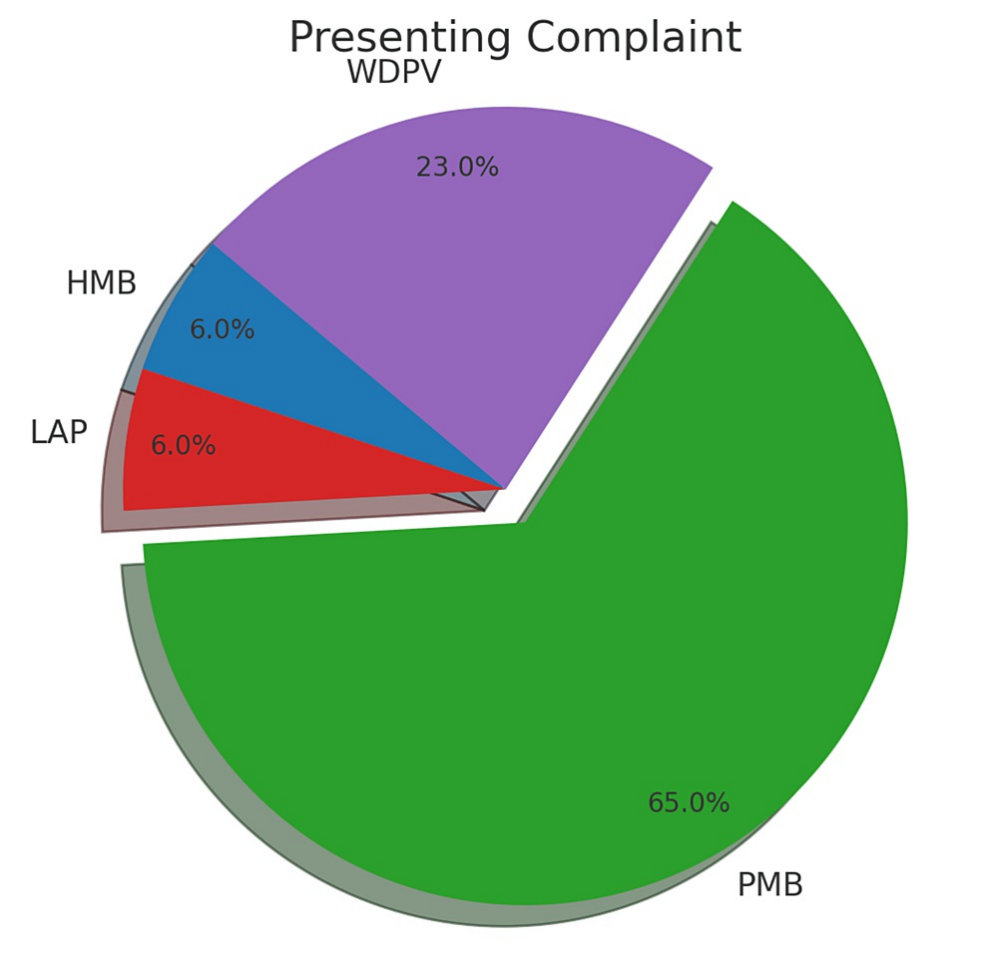
Pie chart showing the breakdown of presenting complaints with postmenopausal bleeding (PMB), accounting for 65% of the presenting complaints. The purple section indicates white discharge per vaginum (WDPV), comprising 23% of the cases. The red slice denotes heavy menstrual bleeding (HMB). The blue slice signifies lower abdominal pain (LAP), each constituting 6% of the reported symptoms.

The majority of the patients were postmenopausal (80.6%) and multiparous (93.5%). The most common presenting complaint was postmenopausal bleeding (64.5%), followed by white discharge per vaginum (22.6%) (Figure 5).

Endometrioid adenocarcinoma was the most common histological subtype (80.6%), followed by clear cell carcinoma (6.5%), serous carcinoma (6.5%), mucinous carcinoma (3.2%), and mixed cell carcinoma (3.2%). Superficial myometrial invasion (<50%) was observed in 19 patients (61.3%), while deep myometrial invasion (≥50%) was seen in 12 patients (38.7%). The distribution of FIGO stages was as follows: stage IA (61.3%), stage IB (22.6%), stage II (9.7%), and stage IIIA (6.5%) (Table 1).

**Table 1:** Histological subtypes of endometrial cancer, the extent of myometrial invasion, and FIGO staging for 31 patients. FIGO: Federation of Gynecology and Obstetrics

Variable	Number of patients with percentages - n(%)
Histological subtype	
Endometrioid	25 (80.6%)
Serous	2 (6.5%)
Mucinous	1 (3.2%)
Clear cell	2 (6.5%)
Mixed cell	1 (3.2%)
Myometrial invasion	
Superficial (<50%)	19 (61.3%)
Deep (>50%)	12 (38.7%)
FIGO stage	
IA	19 (61.3%)
IB	7 (22.6%)
II	3 (9.7%)
IIIA	2 (6.5%)

The accuracy of T2WI, T2WI + DWI, and T2WI + DCE-MRI in assessing superficial myometrial invasion was 61.3%, 87.1%, and 87.1%, respectively (Table 2).

**Table 2:** Diagnostic accuracy of MRI T2-weighted imaging alone, MRI T2-weighted imaging combined with diffusion-weighted imaging (DWI), and MRI T2-weighted imaging combined with dynamic contrast-enhanced (DCE) imaging against histopathological examination (HPE) for assessing superficial myometrial invasion in endometrial cancer, indicating sensitivity, specificity, positive predictive value (PPV), negative predictive value (NPV), and overall accuracy.

			HPE < 50%		Total	Sensitivity	61.1	
			Superficial	Non-superficial		Specificity	61.5	
MRI T2WI < 50%	Yes		11 (35.48%)	5 (16.13%)	16 (51.61%)	PPV	68.8	
	No		7 (22.58%)	8 (25.81%)	15 (48.39%)	NPV	53.3	
Total			18 (58.06%)	13 (41.94%)	31 (100.00%)	Accuracy	61.3	
			HPE < 50%		Total	Sensitivity	88.9	
			Superficial	Non Superficial		Specificity	84.6	
MRI T2WI + DWI < 50%	Yes		16 (51.61%)	2 (6.45%)	18 (58.06%)	PPV	88.9	
	No		2 (6.45%)	11 (35.48%)	13 (41.94%)	NPV	84.6	
Total			18 (58.06%)	13 (41.94%)	31 (100.00%)	Accuracy	87.1	
			HPE < 50%		Total	Sensitivity	88.9	
			Superficial	Non Superficial		Specificity	84.6	
MRI T2WI + DCE < 50%		Yes	16 (51.61%)	2 (6.45%)	18 (58.06%)	PPV	88.9	
		No	2 (6.45%)	11 (35.48%)	13 (41.94%)	NPV	84.6	
Total			18 (58.06%)	13 (41.94%)	31 (100.00%)	Accuracy	87.1	

For deep myometrial invasion, the accuracy of T2WI, T2WI + DWI, and T2WI + DCE-MRI was 64.5%, 90.3%, and 90.3%, respectively (Table 3).

**Table 3:** Comparative analysis of MRI T2WI, MRI T2WI + DWI, and MRI T2WI + DCE in diagnosing ≥50% myometrial invasion against HPE, detailing sensitivity, specificity, PPV, NPV, and accuracy percentages. MRI T2WI: MRI T2-weighted imaging alone, MRI T2WI + DWI: MRI T2-weighted imaging combined with diffusion-weighted imaging, MRI T2WI + DCE: MRI T2-weighted imaging combined with dynamic contrast-enhanced imaging, HPE: histopathological examination, PPV: positive predictive value, NPV: negative predictive value

			HPE >=50%		Total		Sensitivity	66.7	
			Deep	Non-deep			Specificity	63.2	
MRI T2WI >=50%	Yes		8 (25.81%)	7 (22.58%)	15 (48.39%)		PPV	53.3	
	No		4 (12.90%)	12 (38.71%)	16 (51.61%)		NPV	75.0	
Total			12 (38.71%)	19 (61.29%)	31 (100.00%)		Accuracy	64.5	
			HPE >=50%		Total		Sensitivity	91.7	
			Deep	Non-deep			Specificity	89.5	
MRI T2WI + DWI >=50%	Yes		11 (35.48%)	2 (6.45%)	13 (41.94%)		PPV	84.6	
	No		1 (3.23%)	17 (54.84%)	18 (58.06%)		NPV	94.4	
Total			12 (38.71%)	19 (61.29%)	31 (100.00%)		Accuracy	90.3	
			HPE >=50%		Total		Sensitivity	91.7	
			Deep	Non-deep			Specificity	89.5	
MRI T2WI + DCE >=50%		Yes	11 (35.48%)	2 (6.45%)	13 (41.94%)		PPV	84.6	
		No	1 (3.23%)	17 (54.84%)	18 (58.06%)		NPV	94.4	
Total			12 (38.71%)	19 (61.29%)	31 (100.00%)		Accuracy	90.3	

The sensitivity, specificity, PPV, and NPV for T2WI + DWI and T2WI + DCE-MRI were high and comparable (88.9-91.7%) for both superficial and deep invasion. T2WI had markedly lower sensitivity and specificity. The ROC curve analysis demonstrated that T2WI + DWI and T2WI + DCE-MRI had significantly higher diagnostic performance compared to T2WI alone for both superficial and deep myometrial invasion (p < 0.01). For superficial invasion, the area under the curve (AUC) was 0.613 for T2WI, 0.868 for T2WI + DWI, and 0.868 for T2WI + DCE-MRI. For deep invasion, the AUC was 0.649 for T2WI, 0.906 for T2WI + DWI, and 0.906 for T2WI + DCE-MRI (Tables 4, 5)

**Table 4:** Receiver operating characteristic (ROC) curves comparing the diagnostic performance of T2WI, T2WI + DWI, and T2WI + DCE-MRI for assessing superficial myometrial invasion (<50%) in endometrial cancer. The area under the curve (AUC) was significantly higher for T2WI + DWI and T2WI + DCE-MRI compared to T2WI alone (p < 0.01). ** Highly significant at p < 0.01, # no statistical significance at p > 0.05. T2WI: T2-weighted imaging alone, T2WI + DWI: T2-weighted imaging combined with diffusion-weighted imaging, T2WI + DCE-MRI: T2-weighted imaging combined with dynamic contrast-enhanced imaging

Area under the curve				
Test result variable(s)	Area	p-value	95% C.I	
			LB	UB
MRI T2WI < 50%	0.613	0.289 #	.409	.817
MRI T2WI + DWI < 50%	0.868	0.001 **	.724	1.000
MRI T2WI + DCE < 50%	0.868	0.001 **	.724	1.000

**Table 5:** Receiver operating characteristic (ROC) curves comparing the diagnostic performance of T2WI, T2WI + DWI, and T2WI + DCE-MRI for assessing deep myometrial invasion (≥50%) in endometrial cancer. The area under the curve (AUC) was significantly higher for T2WI + DWI and T2WI + DCE-MRI compared to T2WI alone (p < 0.01). ** highly significant at p < 0.01, # no statistical significance at p > 0.05. T2WI: T2-weighted imaging alone, T2WI + DWI: T2-weighted imaging combined with diffusion-weighted imaging, T2WI + DCE-MRI: T2-weighted imaging combined with dynamic contrast-enhanced imaging

Area under the curve				
Test result variable(s)	Area	p-value	95% C.I	
			LB	UB
MRI T2WI >=50%	0.649	0.168 #	.447	.851
MRI T2WI + DWI >=50%	0.906	0.0002 **	.783	1.000
MRI T2WI + DCE >=50%	0.906	0.0002 **	.783	1.000

The chi-square test revealed a highly significant association between the MRI findings and the histopathological stage for T2WI (χ2 = 28.666, p = 0.001), T2WI + DWI (χ2 = 66.696, p = 0.0005), and T2WI + DCE-MRI (χ2 = 78.186, p = 0.0005) (Tables 6-8).

**Table 6:** Comparison of MRI T2WI with the HPE stage by Pearson’s chi-square test MRI T2WI: MRI T2-weighted imaging alone, HPE: histopathological examination. ** highly statistical significance at the p < 0.01 level.

			HPE stage				Total	ꭓ 2 - value	p-value
			IA	IB	II	IIIA			
MRI T2WI	IA	Count	12	3	1	0	16	28.666	0.001 **
		%	38.7%	9.7%	3.2%	0.0%	51.6%		
	IB	Count	7	4	1	0	12		
		%	22.6%	12.9%	3.2%	0.0%	38.7%		
	II	Count	0	0	1	1	2		
		%	0.0%	0.0%	3.2%	3.2%	6.5%		
	IIIA	Count	0	0	0	1	1		
		%	0.0%	0.0%	0.0%	3.2%	3.2%		
Total		Count	19	7	3	2	31		
		%	61.3%	22.6%	9.7%	6.5%	100.0%		

**Table 7:** Comparison of MRI T2WI + DWI with the HPE stage by Pearson’s chi-square test MRI T2WI + DWI: MRI T2-weighted imaging combined with diffusion-weighted imaging, HPE: histopathological examination. ** highly statistical significance at the p < 0.01 level.

			HPE stage				Total	ꭓ 2 - value	p-value
			IA	IB	II	IIIA			
MRI T2WI + DWI	IA	Count	17	1	0	0	18	66.696	0.0005 **
		%	54.8%	3.2%	0.0%	0.0%	58.1%		
	IB	Count	2	6	1	0	9		
		%	6.5%	19.4%	3.2%	0.0%	29.0%		
	II	Count	0	0	2	0	2		
		%	0.0%	0.0%	6.5%	0.0%	6.5%		
	IIIA	Count	0	0	0	2	2		
		%	0.0%	0.0%	0.0%	6.5%	6.5%		
Total		Count	19	7	3	2	31		
		%	61.3%	22.6%	9.7%	6.5%	100.0%		

**Table 8:** Comparison of MRI T2WI + DCE with the HPE stage by Pearson’s chi-square test MRI T2WI + DCE: MRI T2-weighted imaging combined with dynamic contrast-enhanced imaging, HPE: histopathological examination. ** highly statistical significance at the p < 0.01 level.

			HPE stage				Total	ꭓ 2 - value	p-value
			IA	IB	II	IIIA			
MRI T2WI + DCE	IA	Count	17	1	0	0	18	78.186	0.0005 **
		%	54.8%	3.2%	0.0%	0.0%	58.1%		
	IB	Count	2	6	0	0	8		
		%	6.5%	19.4%	0.0%	0.0%	25.8%		
	II	Count	0	0	3	0	3		
		%	0.0%	0.0%	9.7%	0.0%	9.7%		
	IIIA	Count	0	0	0	2	2		
		%	0.0%	0.0%	0.0%	6.5%	6.5%		
Total		Count	19	7	3	2	31		
		%	61.3%	22.6%	9.7%	6.5%	100.0%		

In summary, the current study results demonstrate that the addition of DWI or DCE-MRI to T2WI significantly improves the diagnostic performance of MRI for assessing the depth of myometrial invasion in EC, with comparable accuracy between T2WI + DWI and T2WI + DCE-MRI.

## Discussion

The present prospective study demonstrated that the addition of DWI or DCE-MRI to conventional T2WI significantly improves the diagnostic performance of MRI for assessing the depth of myometrial invasion in EC. Our findings are consistent with previous studies that have highlighted the value of functional MRI techniques in the preoperative staging of EC [11-14].

The depth of myometrial invasion is a crucial prognostic factor in EC, as it correlates with tumor grade, lymph node metastases, and overall patient survival [15]. Accurate preoperative assessment of myometrial invasion is essential for guiding treatment decisions and surgical planning. In the present study, T2WI + DWI and T2WI + DCE-MRI showed high and comparable diagnostic accuracy, sensitivity, specificity, PPV, and NPV for both superficial (<50%) and deep (≥50%) myometrial invasion. The diagnostic performance of various MRI protocols for assessing myometrial invasion in EC across different studies, including the present study, is compared in Table 9.

**Table 9:** Comparison of diagnostic performance of various MRI protocols for assessing myometrial invasion in endometrial cancer across different studies, including our present study.

Study	MRI Protocol	Sensitivity (%)	Specificity (%)	Accuracy (%)
Present study	T2WI	61.1-66.7	61.5-63.2	61.3-64.5
	T2WI + DWI	88.9-91.7	84.6-89.5	87.1-90.3
	T2WI + DCE-MRI	88.9-91.7	84.6-89.5	87.1-90.3
Deng et al. [16]	T2WI	87	58	-
	T2WI + DWI	94	88	-
Beddy et al. [17]	T2WI	68	66	67
	DWI	84	100	90
	DCE-MRI	61	88	71
Bonatti et al. [18]	T2WI	61.1	94.6	85.7
	T2WI + DWI	88.9	91.9	91.1
Neves et al. [19]	Standard MRI evaluation	78.1	84.5	81.6
	Fused T2WI-DWI	85.4	93.5	89.7

The current study results are in line with a meta-analysis by Deng et al. [16], which reported that the combination of DWI and T2WI had higher sensitivity (94%) and specificity (88%) compared to T2WI alone (sensitivity 87% and specificity 58%) for assessing deep myometrial invasion [16]. Similarly, Beddy et al. [17] found that DWI had superior diagnostic accuracy (90%) compared to DCE-MRI (71%) and T2WI (67%) [17]. Bonatti et al. [18] also demonstrated that T2WI + DWI had higher sensitivity (88.9%), specificity (91.9%), and accuracy (91.1%) than T2WI alone (sensitivity 61.1%, specificity 94.6%, and accuracy 85.7%) [18].

Neves et al. [19] conducted a study comparing the diagnostic performance of fused T2WI-DWI images with standard MRI evaluation (including T2WI, DWI, and DCE-MRI) for assessing myometrial invasion in EC. They found that fused T2WI-DWI images had higher accuracy (89.7%), sensitivity (85.4%), and specificity (93.5%) compared to standard MRI evaluation (accuracy 81.6%, sensitivity 78.1%, and specificity 84.5%). These findings further support the added value of combining T2WI and DWI for improved assessment of myometrial invasion.

The improved diagnostic performance of DWI can be attributed to its ability to detect the restricted diffusion of water molecules in highly cellular malignant tumors, which appears as high-signal intensity on high b-value DWI and low ADC values [20]. This restricted diffusion helps to delineate the tumor margins and the depth of myometrial invasion. By contrast, the diagnostic accuracy of T2WI can be limited by factors, such as the presence of adenomyosis, leiomyomas, or tumor extension into the uterine cornua [21]. DCE-MRI, which assesses the perfusion and permeability of tissues, has also been widely used for the preoperative staging of EC [22]. In our study, T2WI + DCE-MRI showed comparable diagnostic performance to T2WI + DWI. However, DWI has several advantages over DCE-MRI, including shorter acquisition times, no need for intravenous contrast administration, and the ability to provide quantitative assessment of tumor cellularity through ADC values [20]. The present study also found a highly significant association between the MRI findings and the histopathological stage for all three MRI protocols (T2WI, T2WI + DWI, and T2WI + DCE-MRI). This highlights the importance of MRI in the preoperative staging of EC, as it can guide the choice of surgical approach (laparoscopic vs. open), the extent of lymph node dissection, and the need for adjuvant therapy [4].

However, the current study has some limitations. First, the sample size was relatively small, which may limit the generalizability of the study findings. Second, the MRI protocols were assessed in a fixed order (T2WI, T2WI + DCE-MRI, T2WI + DWI), which could have introduced a learning bias. Finally, the study did not evaluate the interobserver variability between the two radiologists who assessed the MRI images.

## Conclusions

The present study demonstrates that the addition of DWI or DCE-MRI to T2WI significantly improves the diagnostic performance of MRI for assessing the depth of myometrial invasion in EC. Given the comparable diagnostic accuracy of T2WI + DWI and T2WI + DCE-MRI, and the advantages of DWI over DCE-MRI, incorporating DWI into the standard MRI protocol for the preoperative staging of EC is recommended. DWI may be preferred due to its lack of need for contrast administration and ease of use in clinical practice. Future studies with larger sample sizes and prospective designs are needed to validate the study findings and establish the optimal MRI protocol for EC staging.
